# Artificial Intelligence and Computer Vision in Low Back Pain: A Systematic Review

**DOI:** 10.3390/ijerph182010909

**Published:** 2021-10-17

**Authors:** Federico D’Antoni, Fabrizio Russo, Luca Ambrosio, Luca Vollero, Gianluca Vadalà, Mario Merone, Rocco Papalia, Vincenzo Denaro

**Affiliations:** 1 Unit of Computer Systems and Bioinformatics, Università Campus Bio-Medico di Roma, Via Alvaro Del Portillo 21, 00128 Rome, Italy; f.dantoni@unicampus.it (F.D.); l.vollero@unicampus.it (L.V.); 2 Department of Orthopaedic Surgery, Università Campus Bio-Medico di Roma, Via Alvaro Del Portillo 200, 00128 Rome, Italy; l.ambrosio@unicampus.it (L.A.); g.vadala@unicampus.it (G.V.); r.papalia@unicampus.it (R.P.); v.denaro@unicampus.it (V.D.)

**Keywords:** low back pain, orthopaedics, artificial intelligence, computer vision, digital image processing, deep learning, decision support systems, computer aided diagnosis

## Abstract

Chronic Low Back Pain (LBP) is a symptom that may be caused by several diseases, and it is currently the leading cause of disability worldwide. The increased amount of digital images in orthopaedics has led to the development of methods related to artificial intelligence, and to computer vision in particular, which aim to improve diagnosis and treatment of LBP. In this manuscript, we have systematically reviewed the available literature on the use of computer vision in the diagnosis and treatment of LBP. A systematic research of PubMed electronic database was performed. The search strategy was set as the combinations of the following keywords: “Artificial Intelligence”, “Feature Extraction”, “Segmentation”, “Computer Vision”, “Machine Learning”, “Deep Learning”, “Neural Network”, “Low Back Pain”, “Lumbar”. Results: The search returned a total of 558 articles. After careful evaluation of the abstracts, 358 were excluded, whereas 124 papers were excluded after full-text examination, taking the number of eligible articles to 76. The main applications of computer vision in LBP include feature extraction and segmentation, which are usually followed by further tasks. Most recent methods use deep learning models rather than digital image processing techniques. The best performing methods for segmentation of vertebrae, intervertebral discs, spinal canal and lumbar muscles achieve Sørensen–Dice scores greater than 90%, whereas studies focusing on localization and identification of structures collectively showed an accuracy greater than 80%. Future advances in artificial intelligence are expected to increase systems’ autonomy and reliability, thus providing even more effective tools for the diagnosis and treatment of LBP.

## 1. Introduction

In the last decade, a significant increase in the use of Artificial Intelligence (AI) has been experienced in the most disparate fields, ranging from vocal assistants commonly employed during our daily life to self-driving cars. Thanks to the unique ability of intelligent machines to be trained and automatically acquire new tasks based on previous experience or provided data, the use of AI is being increasingly investigated for applications in medical research [1]. Indeed, AI-based computers have already shown to potentially revolutionize drug design and discovery [2,3], automatic segmentation and relevant data extraction from radiological datasets [4] as well as the formulation of diagnosis, outcome prediction and treatment planning in different medical fields [5,6,7]. The adoption of this ground-breaking technology is being explored in spine surgery as well [1]. Indeed, thanks to its interdisciplinary nature and the wide utilization of radiological images to inspect the anatomical structures of the spine, the use of AI may be of particular value in determining, for example, which are the pathological discs [8], classifying a scoliotic curve [9] and predict its progression [10]. In this study, we have systematically reviewed the available literature on the use of AI, and more specifically computer vision, in the prevention, diagnosis, and treatment of chronic Low Back Pain (LBP).

LBP is mainly caused by intervertebral disc degeneration, and it is currently the leading cause of disability worldwide, as well as the most common reason for workers’ compensation claims [11]. AI has improved the clinical practice with regards to the treatment, prevention and outcome prediction of subjects suffering from LBP. This is mainly due to the ever-growing amount of clinical data available to practitioners, which allow to train and develop increasingly sophisticated AI methodologies. With particular regards to LBP, a huge amount of digital clinical images are gathered daily in order to detect signs of disease in the spinal structures. For this reason, several machine learning algorithms have been developed in recent years in order to speed-up the diagnostic process and to optimize patients’ recovery. The latest AI improvements were accompanied by the outbreak of deep learning and by an increase of computing capacity, which allow to develop models that are getting more and more autonomous and accurate. In particular, computer vision techniques applied to clinical images allow to detect some image features that are invisible to the human eye. The importance of computer vision in relation to LBP is multi-faceted: it allows to perform a plethora of tasks that may improve the clinical practice, such as automatically localizing and detecting lumbar structures with segmentation. Moreover, it allows to extract a set of features from the image that can be used as an input for further machine learning algorithms in order to provide a decision support to the physician or, in other cases, directly suggest the most appropriate diagnosis. For this reason, we have systematically reviewed the available literature on the application of computer vision on the diagnosis and treatment of LBP in order to describe the state of the art of such technology and its potential applications.

## 2. Materials and Methods

In order to perform an exhaustive research of AI articles related to LBP, we performed a query research on PubMed (Query research used: (((Artificial intelligence [Title/Abstract]) OR ((feature extraction[Title/Abstract]) OR ((segmentation[Title/Abstract]) OR (Computer Vision[Title/Abstract]) OR (Machine learning[Title/Abstract])) OR (deep learning[Title/Abstract]) OR (neural network[Title/Abstract]))) AND ((Low Back Pain [Title/Abstract]) OR (lumbar[Title/Abstract]))). All the search words had to be included in the title or in the abstract of the articles: the terms “low back pain” and “lumbar” were considered for the pathological part, and the terms “artificial intelligence”, “feature extraction”, “segmentation”, “computer vision”, “machine learning”, “deep learning” and “neural network” were considered for the AI part. We selected all the articles that included at least one term of the pathological part and at least one term of the artificial intelligence part in their title or abstract.

### 2.1. Inclusion and Exclusion Criteria

The aim of this work was to gather all the works concerning the utilization of AI, and particularly of computer vision, in the diagnosis, prevention, and treatment of chronic LBP and related diseases. Straightforwardly, all the selected articles had to meet all the following inclusion criteria:

• Chronic LBP or lumbar diseases must have been among the main topics of the article. We included works on the prevention, diagnosis or treatment of chronic LBP and treating at least one of the structures involved in LBP (i.e., vertebrae, discs, muscles);
• AI must have been used in the work with application to clinical images. We included articles exploiting AI methods falling in the areas of computer vision, machine learning and artificial Neural Networks (NNs);
• Subjects of the study: all the articles must have been based on studies of human low back and related pathology, regardless of the age or employment of the subjects included in the study;
• Language: all articles must have been written in English.

Conversely, articles that were excluded did not meet the inclusion criteria for one of the following reasons:

• A different medical problem was considered: we excluded articles which did not consider chronic LBP and its related physical structures and medical data. For example, we excluded studies that considered only cervical or thoracic vertebrae, or that focused on osteoporosis, metastases, traumatic LBP, and other causes of non-discogenic LBP;
• AI was not considered: some articles in the search results proposed definitions and practice for LBP based only on medical observation without utilization of AI;
• Computer vision and clinical images were not considered in the study, regardless of whether AI was utilized for developing diagnosis or support systems;
• Animal studies: we excluded studies based on vertebral structures of animals;
• Embryonal studies: we excluded studies performed on embryos and concerning the embryogenesis of spinal structures.

**Figure 1:**
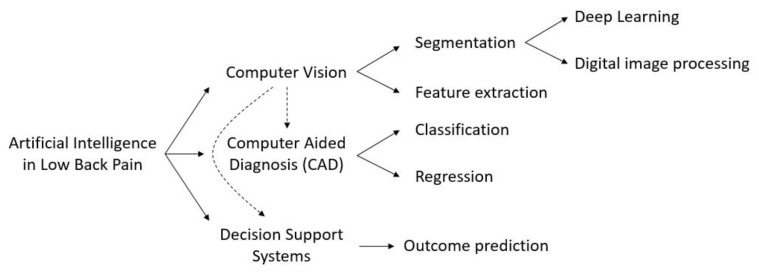
Schematic partitioning of the works concerning the application of AI in LBP.

A preliminary screening of the article selection allowed us to define three main categories in which the utilization of AI in LBP might be split, namely computer vision, computer aided diagnosis, and decision support systems (DSSs) (Figure 1). Computer vision is the field of AI that deals with how computers can gain high-level understanding from digital images or videos. With regards to LBP, its main applications concern feature extraction and image segmentation. Feature extraction is a dimensionality reduction process which is applied to images obtained using Magnetic Resonance Imaging (MRI), ultrasound, X-rays, and Computed Tomography (CT). The main goal of feature extraction is to retrieve a restricted number of relevant features from an image without losing important information, in order to facilitate subsequent tasks such as classification or regression.

Image segmentation is the task of dividing an image into subregions corresponding to different elements of the image. More in depth, the goal of image segmentation is the labeling of each pixel of an image with a corresponding class, e.g., foreground or background, in order to detect the relevant elements of an image. It mainly resorts to two principal techniques: deep learning, in which the image is directly given as input to an artificial NN which is trained on other images to automatically identify subregions, and digital image processing (DIP) techniques, which process digital images to find the edges of different regions based on semantic characteristics, exploiting methods such as gradient thresholding or statistical shape models.

Computer aided diagnosis is a group of techniques which help medical practitioners in identifying a pathology or in quantifying the grade of a disease. It can be split into classification and regression, in which machine or deep learning models are used to assign a predefined label or to generate a numeric output, respectively. In practice, classification is used to identify or categorize a pathology, whereas regression is used to produce a quantitative evaluation of some measure.

Decision support systems (DSSs) are software systems that allow medical practitioners to enhance the decision making and improve the outcome of patients suffering from a specific disease. The goal of the vast majority of DSSs is the outcome prediction, i.e., the prediction of the improvement that a patient would experience after exposure to a defined therapy. By predicting the extent to which a patient would benefit from a specific treatment, DSSs provide the physician with practical tools to assess whether or not surgery may be preferable to conservative treatment. Finally, DSSs can be used for prevention, e.g., by providing the user with recommendations or correct practice for preventing the onset of a disease. It is worth noting that computer vision techniques can be used as preprocessing for developing a DSS, as well as a for computer aided diagnosis.

### 2.2. Evaluation Metrics

Different tasks use different metrics to evaluate the performance of AI systems. However, considering the large amount of works reported in this review, different metrics were also considered within the same task. With regards to the feature extraction task, no specific evaluation metric was considered. This is because, in most cases, feature extraction is exploited as a preliminary step for further tasks such as classification and regression, and most papers only report the performance for the latter.

With regards to the classification task, we reported the results in terms of accuracy (Acc), where available. For brevity purposes, let us consider a binary classification task, e.g., positive vs. negative. Given a test set composed of *N* samples, defined the True Positives (TP) as the number of positive samples correctly classified, and the True Negatives (TN) as the number of negative samples correctly classified, then accuracy is defined as:

(1) $$Acc\%=\frac{TP+TN}{N}\times 100$$

Thus, greater values correspond to a better performance. For each class, recall and precision can be computed as well. Defined the False Positives (FP) and False Negatives (FN) as the number of misclassified positive/negative samples, then recall and precision are computed as:

(2) $$Recall=\frac{TP}{TP+FN}\;Precision=\frac{TP}{TP+FP}$$

In binary problems, recall is also called True Positive Rate and corresponds to sensitivity, whereas the True Negative Rate is also called specificity. In the case of multi-class problems, accuracy is computed by considering the TP for each class, and recall and precision per class can be computed. For imbalanced datasets, the F1-Score can be computed for each class. The F1-Score for class *c* is defined as:

(3) $$F1-Score_{c}=\frac{2\cdot Recall_{c}\cdot Precision_{c}}{Recall_{c}+Precision_{c}}$$

and takes into account both recall and precision of the class. Another widely used evaluation metric is the Area Under the Curve (AUC), which corresponds to the area under the Receiver Operating Characteristic (ROC) curve showing the performance of a classification model at all classification thresholds, which is plotted considering the True Positive Rate against the False Positive Rate. Its values range from 0 to 1 (the closer to 1, the better the performance).

With regards to the regression task, let us consider a sequence of original values x(t) and a sequence of predicted values $\tilde{x}\left(t\right)$. The Mean Absolute Error (MAE) for a sequence of *N* timestamps is defined as:

(4) $$MAE=\sum_{t=1}^{N}\frac{|x\left(t\right)-\tilde{x}\left(t\right)|}{N}\;$$

Thus, the closer to 0 the value, the better the performance. In some cases, percentage error values are used to evaluate performance, the meaning of which varies with the investigated task.

With regards to the segmentation task, two main percent performance indices are used which evaluate to what extent the segmentation result is close to the desired segmentation. As stated, segmentation consists in labeling each pixel of an image. Given two sets of data *A* and *B*, corresponding to the desired and the effective segmented areas, the Sørensen–Dice coefficient (DICE) is defined as:

(5) $$DICE\left(A,B\right)=\frac{2\cdot |A\cap B|}{\left|A|+|B\right|}$$

where |A| and |B| are the cardinalities of the two sets. It divides the number of common elements of the two sets by the total number of elements of the two sets. When applied to binary data, it is equivalent to the *F1-Score*. Differently, the Jaccard index is defined as:

(6) $$Jaccard\left(A,B\right)=\frac{\left|A\cap B\right|}{\left|A\cup B\right|}$$

and is also known as Intersection Over Union. For both indices, the closer to 100% the value, the better the performance. It is worth noting that DICE(A,B)≥Jaccard(A,B) for any couple of sets (A,B), and the relation Jaccard=DICE/(2−DICE) exists to compute one value from the other.

## 3. Quality of Evidence

**Figure 2:**
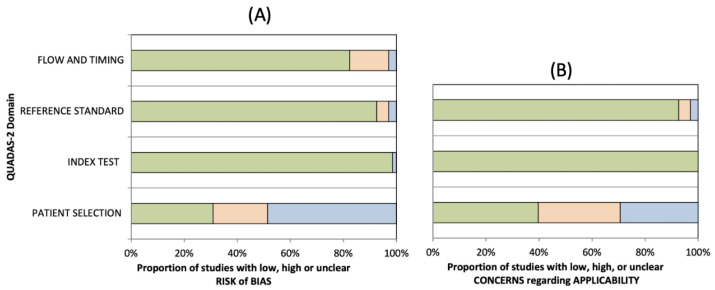
Summary of the methodological quality of included studies regarding the 4 domains assessing the risk of bias (**A**) and the 3 domains assessing applicability concerns (**B**) of the QUADAS-2 score. The portion of studies with a low risk of bias are highlighted in green, studies with an unclear risk of bias are depicted in blue and studies with a high risk of bias are represented in orange.

The methodological quality of the included studies was graded independently by two reviewers (L.A. and F.R.), and any disagreement was resolved by the intervention of a third reviewer (G.V.) The risks of bias and applicability of the included studies were assessed by using customized assessment criteria based on the Quality Assessment of Diagnostic Accuracy Studies (QUADAS-2) [12]. This tool is based on 4 domains: patient selection, index test, reference standard, and flow and timing. Each domain is evaluated in terms of risk of bias, and the first 3 domains are also assessed in terms of concerns regarding applicability. Sixty-eight studies were rated on a 3-point scale, reflecting concerns about risk of bias and applicability as low, unclear or high, as shown in Figure 2 (the details of analysis are presented in Tables S1 and S2).

## 4. Results

**Figure 3:**
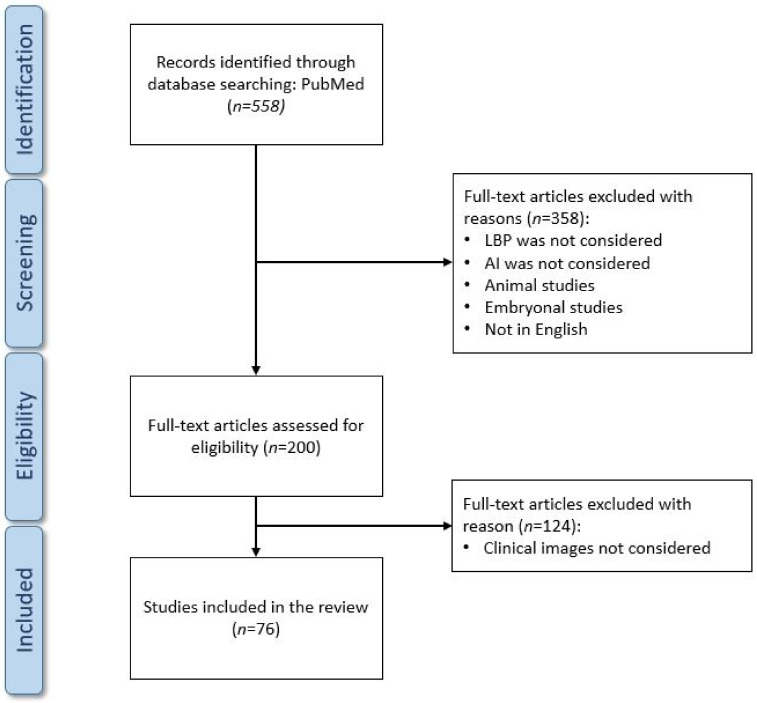
Preferred Reporting Items for Systematic reviews and Meta-Analyses (PRISMA) flow diagram.

The search was performed on 18 March 2021, and resulted in 558 articles. Nonetheless, many of these articles focused on a different topic from that of this review, so after a first screening based on the article titles and abstracts we reduced the number of eligible articles to 200. A second screening phase was performed after having read the full text of each article, which led the total amount of included articles to 76. We created a flow-chart diagram according to the PRISMA protocol that shows the selection process of the studies (Figure 3). The articles were screened by two independent reviewers and, in the event of discrepancies regarding the inclusion or exclusion of an article, they discussed together until consensus was reached.

It is worth noting how the amount of published work is increasing year by year, and that the number of articles published in 2020 is almost double that of 2019. This may be due to two main reasons: first, the ever-increasing amount of clinical images and data available to researchers and, secondly, the improvement of computing capacity observed in recent years. The final results of the search also include five reviews. One of them, published in 2020 by Tagliaferri et al. [13], is specifically focused on LBP, but considers only the diagnosis and prognosis capability of AI in comparison with the McKenzie and the STarT Back methods, and without taking into account works that exploit clinical images. The other four reviews do not focus specifically on LBP. In detail, in 2019 Tack [14] focused on musculoskeletal medicine in general, and determined in which fields AI had reached human prediction levels; in 2020, Azimi et al. [15] focused on the use of NNs for the treatment of the whole spine; in 2019, Galbusera et al. [1] described the application of AI to problems related to the whole spine; finally, in 2016 Yao et al. [16] performed a multi-center milestone comparative study for vertebral segmentation methods based on CT images. Two articles presenting databases were also found: LUMINOUS, which is a database of ultrasound images from 109 patients for multifidus muscle segmentation [17], and MyoSegmentum, which includes MRI images of 54 patients for the segmentation of lumbar muscles and vertebral bodies [18].

The remainder of this section reports the results of the search that include works concerning computer vision. In particular, we have listed manuscripts that performed a feature extraction task or that performed semantic segmentation, and we have described papers that used DIP/NN approaches in two different subsections.

### 4.1. Feature Extraction

Feature extraction is a dimensionality reduction process aimed at identifying a restricted set of relevant features in order to improve the predictive capability of a system. In this review, we identified a total of 8 papers, whose main characteristics are reported in Table 1, aiming to extract relevant features from several types of LBP-related images. In detail, we included:

• six articles on MRI (1 of which considers 3D MRI);
• one article on 3D images of the back surface;
• one article on X-ray imaging.

Intervertebral discs (IVDs) are the most investigated lumbar structures (five papers), followed by vertebrae (three papers), whereas one paper evaluated LBP without focusing on a specific structure. It is worth noting that only two out of eight articles have exclusively focused on feature extraction, i.e., the work of Raudner et al. [21] in which the GRAPPATINI method is presented for IVD feature extraction from MRI, and the work of Abdollah et al. [22] in which a Random Forest and a Texture analysis are exploited on MRI for feature extraction from IVDs and vertebrae, respectively. The remaining six articles described the performance of further tasks after feature extraction. In detail, four of them performed classification, one performed regression, and one performed segmentation tasks.

All the works that performed further tasks following feature extraction exploited machine learning techniques rather than deep learning: this is one of the advantages of feature extraction, as it allows to achieve results using much faster and less computationally-expansive methods. With regards to classification, Adankon et al. [19] were the only ones to use 3D images of the surface of the human back: they extracted features for 165 patients using local geometric descriptors, and fed them to a least-squares Support Vector Machine (SVM) for the classification of scoliosis curve types, achieving 95% accuracy. Yang et al. [8] used a Gabor wavelet transform to extract features from MRI of 109 subjects, and a Kanade–Lucas–Tomasi (KLT) feature tracker to identify lumbar degenerative changes with an accuracy of 88.3%. Ruiz-España et al. [23] extracted features from MRI of 67 patients using Gradient Vector Flow, and tested several machine learning models to classify degenerated IVDs achieving accuracies greater than 90%. Ketola et al. [24] performed texture feature extraction from 518 MRI and used Logistic Regression to discriminate between symptomatic and asymptomatic LBP with an accuracy of 83%.

With regards to the regression task, Garcia-Cano et al. [10] extracted features from X-ray images of 150 patients through the medium of Independent Component Analysis, and used Random Forest Regression to predict the spinal curve progression in adolescents with idiopathic scoliosis, achieving a MAE of 4.79° for the Cobb angle.

With regards to the segmentation task, Castro-Mateos et al. [20] extracted features from 3D MRI of 59 subjects and performed IVDs segmentation using statistical shape model space and B-Spline space, achieving an average DICE score of 88.4%.

### 4.2. Segmentation

Image segmentation is the task of dividing an image into sub-regions corresponding to different elements of the image, with the aim of accurately identifying the borders of different elements in the image. This approach usually exploits manually-segmented images to train an AI model. Several manuscripts included in the reviewed performed a segmentation task, and some used segmentation as a preliminary step for further tasks. For this reason, in the next sections we report, where applicable, not only the segmentation results, but also those of the successive tasks for which segmentation is used with the aim of localizing and/or identifying structures. In this review, we refer to the task of detecting specific components (e.g., vertebrae) as “localization”, whereas we refer to the task of assigning a label to specific components (e.g., L1, L2, etc.) as “identification”. Moreover, we have differentiated included papers based on whether they exploited DIP techniques or NNs. In this review, we identified 38 manuscripts using DIP techniques, and 23 using NNs. However, it is worth noting how most recent research efforts are moving towards deep learning techniques: taking into account the articles published in the last 5 years (2016-2021), this review includes 16 papers using DIP, and 23 using NNs.

#### 4.2.1. Digital Image Processing

DIP segmentation techniques process digital images to find the edges of different regions based on semantic characteristics, exploiting methods such as gradient thresholding or statistical shape models. In this review, we identified a total of 38 papers that performed DIP segmentation on different types of images (Table 2):

• 15 articles on MRI (2 of which considered 3D MRI);
• 15 articles on CT images;
• 1 articles on both MRI and CT images;
• 3 articles on fluoroscopic images;
• 2 articles on ultrasound images;
• 2 articles on X-ray images.

Vertebrae are the most investigated lumbar structures (26 papers), followed by IVDs (10 papers) and muscles (6 papers). It is worth noting that only one [25] out of the 21 works using CT, X-ray or fluoroscopic images did not involve segmentation of vertebral structures. In total, 20 articles focused only on segmentation without further tasks. Among the others, 12 performed successive structure localization, 6 conducted successive structure identification (4 of which performed both localization and identification), whereas regression, tracking, and 3D reconstruction were investigated by 1 manuscript for each task, respectively.

With regards to the papers that focused exclusively on segmentation, Haq et al. [26] used shape-aware models on 3D MRI of 21 patients for the segmentation of IVDs, achieving an average DICE of 91.7%. In addition, in a successive article Haq et al. [25] utilized a shape statistical deformable model for the segmentation of IVDs on CT images of 18 subjects from the SpineWeb dataset, achieving DICE scores ranging from 91.7 to 95.4%. Li et al. [28] applied a threshold to the results of a Gaussian Mixture Model for segmenting vertebrae on a total of 115 CT images from the SpineWeb and the Microsoft Research datasets, with an average DICE of 92.1%. Ibragimov et al. [29] used landmark detection and deformable models for segmenting 30 vertebrae on CT images, with a DICE of 84.7%. Yu et al. [30] utilized bone-sheet assisted grid cut to segment vertebrae from 21 CT images, achieving an average DICE of 93.9%. Korez et al. [31] applied a shape-constrained deformable model for vertebrae segmentation from CT images of 220 patients, with a DICE of 94.6%. Al-Helo et al. [32] combined Active-shape models and GVF-snake for the segmentation of vertebrae from CT images of 50 subjects, assessing the segmentation quality by visual evaluation. Ruiz-España et al. [33] used a Selective Binary Gaussian Filtering Regularized Level Set to segment vertebrae on CT images of 10 subjects, achieving an average DICE of 95%. Huang et al. [34] exploited Otsu thresholding, Edge- and Region-based level sets to segment vertebrae on CT images of 56 subjects, with a 94% DICE. Mastmeyer et al. [38] utilized volume growing and morphological operations to segment vertebrae on CT images of 41 subjects, achieving DICE scores greater than 98.6%. Zhang et al. [53] applied Hough transform and Fourier descriptors for vertebrae segmentation on one fluoroscopic image, assessing the segmentation quality by visual evaluation. Michopoulou et al. [45] used an Atlas-robust-fuzzy C-Means for segmenting IVDs on MRI of 34 subjects, achieving a 90% DICE. Fallah et al. [46] exploited Hierarchical Conditional Random Fields and a Random Forest for the segmentation of IVDs and vertebrae, respectively, on MRI of 34 subjects, achieving a DICE of 92.5 and 91.4%, respectively. Ghosh et al. [47] combined Random Forest and context features for the segmentation of IVDs and vertebrae, respectively, on MRI of 212 subjects, achieving a DICE of 87 and 84%, respectively. Kim et al. [48] used graph-based and line-based segmentation algorithms for segmenting vertebrae on MRI of 19 patients, achieving a 90% DICE. Gaonkar et al. [49] applied a multi-parametric ensemble to segment vertebrae on MRI of 63 subjects, with an average DICE of 83%. Gawel et al. [50] combined a cascade classifier and an Active Appearance Model to segment vertebrae on 50 MRI, achieving a DICE of 91.4%. Engstrom et al. [51] used a Statistical Shape model for the segmentation of the quadratus lumborum muscle on MRI of 20 patients, achieving a DICE of 87%. Baum et al. [52] exploited an Average Shape model and a Dual Feature model for paraspinal muscle segmentation on MRI of 10 subjects, with a DICE of 83%. Jurcak et al. [54] applied Probabilistic atlases and Geodesic Active Contours for the segmentation of quadratus lumborum muscle on MRI of 20 subjects with a 77% DICE. Ribeiro et al. [61] used Gabor Filters and an ANN to segment vertebrae on X-ray images of 41 patients, achieving a DICE of 91.7%.

With regards to the articles that performed localization following segmentation, Mahdy et al. [35] used a threshold method followed by an adaptive K-Means for the segmentation and localization of lumbar vertebrae on CT images of 10 subjects in order to identify degenerated IVDs, and evaluated the performance by visual evaluation. Courbot et al. [36] exploited a Hidden Markov Chain for semi-automated segmentation of vertebrae on CT images of 15 subjects, achieving a localization accuracy of 89.4%. Rasoulian et al. [37] developed a multi-object shape model for vertebrae localization on 32 CT images, correctly localizing the centers of mass with a MAE of 2 mm with the aim of identifying the optimal location for spinal needle injection. Štern et al. [42] performed an analysis of the geometry of the spinal structures to localize the centers of IVDs and vertebrae on 13 MRI and 29 CT images, respectively, with a localization error of 2.8 and 1.8 mm, respectively. Neubert et al. [56] used an Active Shape model to segment IVDs on MRI of 44 subjects achieving a DICE of 92.3%, and an AUC of 0.98 for localization of degenerated IVDs using Linear Discriminant Analysis and SVM. Kim et al. [59] exploited Fuzzy C-Means Clustering for the localization of lumbar multifidus muscle on ultrasound images of 50 subjects, with a 2 mm localization discrepancy. Lui et al. [60] utilized Decoupled Active Contour for the localization of lumbar multifidus muscle on ultrasound images of 10 subjects, achieving an F1-Score of 90.9%. Sa et al. [62] used Gradient Vector Flow Snake and SVM for the localization of vertebrae on X-ray images of 30 subjects, achieving a True Positive Rate of 75%.

With regards to the papers that performed identification following segmentation, Neubert et al. [27] used a Statistical Shape model on 3D MRI of 28 subjects to segment and identify IVDs and vertebrae, achieving segmentation DICE of 89 and 91%, respectively, and 98.3% specificity and 100% sensitivity for the identification of degenerated IVDs. Castro-Mateos et al. [58] described an Active Contour Model for the segmentation and a Feedforward NN for the identification and classification of IVDs on MRI of 48 subjects, achieving 87% Sensitivity.

With regards to the papers that performed both localization and identification, Jimenez-Pastor et al. [39] used a Decision Forest and morphological image processing to localize and identify vertebrae on 272 CT images, achieving a localization error of 13.7 mm and an accuracy of 74.8%. Lee et al. [40] exploited threshold and thinning-based integrated cost on CT images of 19 subjects, for the localization and identification of lumbar pedicles in order to increase accuracy and safety during transpedicular screw placement, with a localization error of 0.14 mm and 93.2% accuracy. Klinder et al. [41] used a Triangulated Shape model on CT images of 64 subjects, achieving a vertebrae localization error of 1.1 mm and 92% accuracy. Oktay et al. [57] combined a Probabilistic model with an SVM to localize and detect IVDs on MRI of 40 subjects, achieving a localization rate of 95.4% and an accuracy of 97%.

In addition, Wong et al. [43] used Wavelets and a Shape-Active Contour-Based model for vertebrae segmentation and Tracking on 2 videos of fluoroscopic images, evaluating the performance by visual evaluation. Zheng et al. [44] utilized Statistical Shape models for vertebrae segmentation and 3D reconstruction on 4 fluoroscopic images, achieving a mean reconstruction error of less than 1.6 mm. Finally, Fortin et al. [55] used a threshold algorithm for segmentation and quantification of paraspinal muscle composition with a reliability coefficient ranging between 97 and 99%.

#### 4.2.2. Deep Learning

Deep learning is a class of AI algorithms based on Artificial Neural Networks. More in detail, an NN is said to be “deep” if it is composed of more than 2 hidden layers. Deep learning techniques for segmentation take as an input the whole original image, and perform feature extraction, feature selection, segmentation and any further step (e.g., classification, regression) in one single model. In this review, we identified a total of 23 papers that performed deep learning segmentation, and their main characteristics are reported in Table 3. In detail:

• 13 articles on MRI (2 of which considered 3D MRI and 1 with the addition of clinical notes);
• 5 articles on CT images;
• 4 articles on X-ray images (1 of which in combination with Moire images);
• 1 article on ultrasound images.

Vertebrae were the most investigated lumbar structures (16 papers), followed by IVDs (11 papers), spinal canal (7 papers), and muscles (5 papers). In total, 9 articles focused exclusively on segmentation without further tasks. Among the others, 5 manuscripts performed successive structure identification, 3 carried out a regression task, 3 performed successive structure reconstruction, 1 work performed classification, 1 performed structure localization, and 1 carried out both structure localization and identification. It is worth noting that the vast majority of the works included in this section exploited Convolutional Neural Networks (CNNs) or models that derive from them.

With regards to the articles that focused exclusively on segmentation, Iriondo et al. [63] used a Coarse-to-fine context memory NN to segment IVDs on 3D MRI of 31 subjects, achieving a DICE greater than 85%. Malinda et al. [67] utilized Generative Adversarial Networks (GANs) for vertebrae segmentation on CT images of 120 subjects, achieving a DICE of 94.2%. Kim et al. [71] exploited a BSU-net for IVDs segmentation on 20 MRI from the SpineWeb dataset, achieving a DICE of 89.4%. Shen et al. [72] used a Feedforward NN on MRI of 120 subjects, achieving a Jaccard index for the segmentation of IVDs, spinal canal and muscles of 87, 82 and 85%, respectively. Gaonkar et al. [73] applied a U-net to segment IVDs on 39295 MRI images, achieving an 88% DICE; they also combined an SVM with a Regression Tree to segment the spinal canal with a DICE of 87%. Huang et al. [74] used a U-net to segment IVDs and vertebrae on 100 MRI achieving a Jaccard index of 92.6 and 94.7%, respectively. Li et al. [75] utilized a CNN to segment vertebrae and spinal canal on MRI of 120 patients achieving an overall DICE of 92.5%. Moreover, they used a deformed U-net [76] for the segmentation of paraspinal muscles on 120 MRI achieving an overall DICE greater than 91.3%. Zhou et al. [77] utilized a U-net for vertebrae segmentation on MRI of 57 subjects, achieving a DICE of 84.9%.

With regards to the papers that performed structure identification following segmentation, Siemionow et al. [68] used a CNN to identify vertebrae on CT images of 45 subjects, with an overall accuracy ranging from 96 to 99%. Zhou et al. [80] combined a CNN and similarity with a beforehand lumbar image for vertebrae identification on MRI images of 1318 healthy and unhealthy subjects, achieving an accuracy of 98.9%. Forsberg et al. [81] combined a CNN and graph-based graphical models on MRI enriched with clinical notes to identify vertebrae of 475 patients, achieving an accuracy of 97%. Baka et al. [82] utilized a CNN and a matching strategy for vertebrae identification on ultrasound images from 19 datasets, achieving an accuracy of 92%. Li et al. [84] were the only to perform vertebrae identification on X-ray images. They applied a CNN on 110 images, achieving an 80.4% accuracy.

With regards to the articles that performed a regression task, Watanabe et al. [70] used a CNN to estimate spinal alignment on 1996 Moire images, with a Cobb angle MAE of 3.42°. Natalia et al. [79] combined a SegNet and a Contour Evolution Algorithm to measure anteroposterior diameter and foraminal widths on MRI of 515 patients suffering from lumbar spinal stenosis with a mean error of 0.9 mm. Cho et al. [83] used a U-net for the automated segmentation and measurement of lumbar lordosis on X-ray images of 629 patients, achieving a DICE of 82.1% and a MAE of 8.06°.

With regards to the articles performing a Reconstruction task, Staartjes et al. [64] developed a CNN to segment and reconstruct the lumbar structures from 3D MRI of 3 patients, evaluating the performance by visual evaluation. Lee et al. [65] used GANs to generate synthetic spine lumbar structures MRI from 280 CT images, with a MAE of 21 pixels. Fan et al. [66] axploited a U-net to reconstruct lumbar structures from CT images of 108 subjects, with a Kambin triangle of 161 mm${}^{2}$.

With regards to the articles performing a classification task, Jamuladin et al. [78] used a CNN for classification of IVDs and vertebrae on MRI of 2009 subjects achieving an accuracy of 95.6%.

In addition, Sa et al. [85] fine-tuned a Faster Region-based CNN (R-CNN) for IVD localization on 1081 X-ray images with a 90.5% precision. Finally, Netherton et al. [69] used an X-net ensemble to localize and identify vertebrae on 330 CT images, achieving a localization error of 2.2 mm and an accuracy of 94%.

## 5. Discussion

Due to the extensive use of advanced imaging modalities and the complexity of anatomical structures involved in the development of LBP and its sequelae, a vast body of research has been investigating the utilization of AI in the elaboration of digital images for different purposes. The vast majority of the works in the literature exploit MRI or CT imaging, whereas a minority of works exploit X-ray, fluoroscopic or ultrasound imaging. It is worth noting that vertebral structures are the main focus of articles performing segmentation, both with DIP and deep learning techniques; conversely, articles performing feature extraction are mainly focused on IVDs.

With regards to feature extraction, which is the capacity of a system to recognize a specific set of relevant features, all included studies collectively showed an accuracy > 80% in identifying the location of vertebrae [24] and IVDs [8,20,21,22,23,24], with the ability to even detect annular tears and lumbar disc herniation [21,22,23]. Although the majority of the studies were conducted on MRI images [8,20,21,22,23,24], one study utilized X-ray imaging [10] and another study built a 3D model of patients’ backs using a noninvasive surface acquisition technology [19]. Moreover, some of these studies also reported the capacity of the described systems to perform classification and regression tasks on extracted data, such as estimating the degree of IVD degeneration [8,22,23,24], scoliosis curve type classification [19] and prediction of curve progression [10], the presence of spinal stenosis [23] and to explore the correlation between degenerative changes and the presence of LBP [24].

However, most studies focused on segmentation, which is the differentiation of specific subregions of an image based on distinct parameters. Traditionally, segmentation tasks have been performed by DIP systems via subdivision of elements within an image based on gradient thresholding or statistical shape models, which fall under the definition of semantic segmentation [86]. However, recent research has been exploring the use of deep learning-based AI systems which are able to perform multiple tasks at the basic and advanced level in a single model [1]. Vertebrae are by far the most investigated structure, with AI systems reaching > 90% DICE and > 90% accuracy in the majority of studies included in our review, both using DIP [28,29,30,31,32,33,34,35,36,37,38,39,40,41,43,44,48,49,50,53,61,62] and deep learning models [67,69,77,80,81,82,83,84]. In particular, a study from Lee et al. [40] proposed a model to obtain an automated segmentation of lumbar pedicles from CT images in order to increase accuracy and safety during transpedicular screw placement. On the other hand, a study from Watanabe and colleagues [70] described a CNN able to estimate spinal alignment, vertebral rotation and Cobb angle with a mean absolute error of 3.6 pixels for vertebral position, 2.9° for vertebral rotation and 3.42° with regards to the estimated Cobb angle. Similarly, Cho et al. [83] presented a CNN capable of segmenting lumbar vertebrae and subsequently calculate lumbar lordosis, with a mean absolute error of 8.055°. In this manuscript, Several AI systems for automated segmentation of IVDs have been described as well [25,26,45,48,56,57,58,63] with a reported DICE > 90% in nearly all studies. Besides, performance of systems developed for the segmentation of paraspinal muscles have reported a higher variability compared to other structures [51,52,54,55,60,71], with higher DICE values for systems based on deep learning models [76]. In addition, some studies evaluated the simultaneous segmentation of multiple structures, in particular IVDs and vertebrae [27,42,46,56,74,78], with a DICE > 90% in DIP-based systems [27,42,46,56] and a reported accuracy > 95% in most deep learning-based systems [68,71,74,78,85]. Furthermore, some of the latter have been used in order to synthesize CT images from MRI and vice versa. For example, Staartjes et al. [64] introduced a CNN-based system able to generate synthetic CT images from spine MRI, so as to acquire more precise information about osseous structures compared to traditional MRI without the need to expose patients to additional radiation. On the other hand, Lee and colleagues [65] presented a model based on GANs capable of producing a synthetic MRI from spine CT scans, which resulted in a mean overall similarity with real MRI scans of 80.2%. This study demonstrated the possibility to extract accurate information about soft tissues from spine CT without the necessity to order an MRI, which is often expensive and time-consuming. Other studies have also shown the possibility to automatically calculate the spinal canal area [73] as well as segmenting and reconstructing multiple structures at the same time [47,66,72,75,79] with an elevate degree of accuracy.

**Figure 4:**
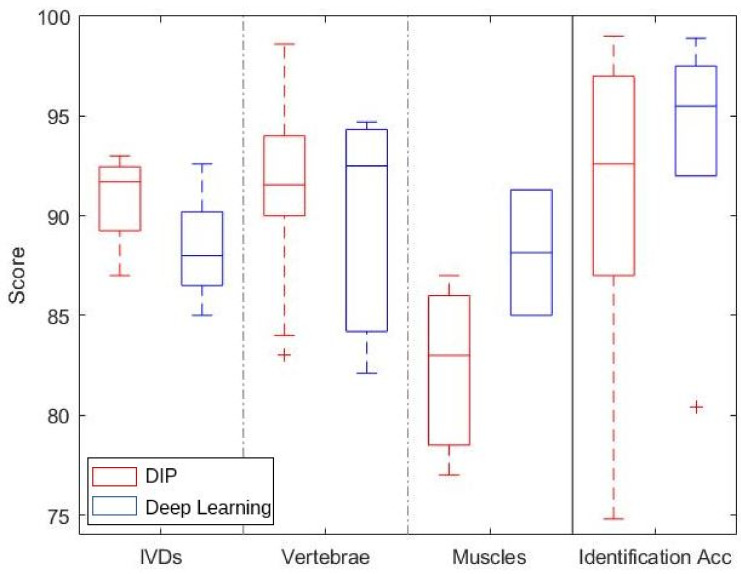
Boxplot summarizing the results for different structures and tasks. The three left columns refer to the DICE scores for the segmentation of IVDs, vertebrae and muscles; the right column refers to the identification accuracy for different structures.

Figure 4 shows a boxplot that summarizes the results for the segmentation of IVDs, vertebrae and lumbar muscles, and the identification accuracy for different lumbar structures. With regards to the segmentation of IVDs and vertebrae, it is worth noting that DIP and deep learning techniques achieve very similar results, with DIP methods performing slightly better. This is mainly due to the regular and homogeneous surface of such structures, whose well-defined edges can be effectively identified using DIP techniques such as threshold and region-growing methods. Conversely, lumbar muscle segmentation performance of deep learning techniques is sensitively better than that of DIP methods. Indeed, the structure of muscles is irregular and more challenging to detect properly, and deep NNs provide a better tool for such a task. With regards to the identification accuracy, deep learning provides generally better results; nonetheless, DIP methods followed by machine learning techniques are typically faster and less computationally expensive, and, in some cases, provide similar performance.

Although the application of computer vision to the elaboration of radiological images of the spine is continuously increasing, some concerns still exist. Indeed, system validation still largely depends on multiple user interventions and cannot replace the human counterpart for obvious reasons, from both clinical and ethical perspectives. Furthermore, the best performing methods are based on the application of NNs, which usually require a large amount of images and computational capacity for training, which are not available to all researchers. However, some DIP techniques provide equal or better performance in the segmentation of regular-shaped structures such as vertebrae and IVDs, while requiring a smaller amount of data for training and limiting the computational burden. Moreover, some methods already exist for the automatic detection and grading of conditions such as spondylolisthesis, disc herniation and scoliosis.

## 6. Conclusions

In the last decade, the utilization of AI has increased considerably in all fields, and medical research made no exception. Indeed, AI-based computers have already shown the potential to revolutionize the medical field, including spine surgery. In this study, we have systematically reviewed the available literature on the use of AI, and more specifically computer vision, in the prevention, diagnosis, and treatment of LBP. In conclusion, computer vision techniques bear promises for effectively improving clinical practice in coming years, thanks to the availability of public datasets and to the natural upcoming increase of the computational capacity. Furthermore, steps are being taken towards the interpretability of AI and, in particular, of deep learning models. Such improvements will lead to the development of systems that will not require multiple user interventions, thus providing a valid assessment tool for physicians. LBP diagnosis and treatment often require the utilization and integration of advanced imaging modalities. In addition, several structural alterations, often subtle and nonunivocal to interpret, concur to define the clinical scenario. In this picture, the use of AI and computer vision may effectively assist and implement the diagnostic process, thus possibly improving clinical outcomes and diagnostic accuracy.

## Figures and Tables

**Table 1:** Feature extraction. For each work, it is reported whether or not other tasks are performed following feature extraction. The reported results are related to the task following feature extraction. Abbreviations are used for Magnetic Resonance Imaging (MRI), Low Back Pain (LBP), Accuracy (Acc), Mean Absolute Error (MAE), Machine Learning (ML), Support Vector Machine (SVM).

Author/Year	Main Task	Data Type	# Patients	Structures Involved	Results	Model
Adankon, 2012 [19]	Feature Extraction and Classification	3D image of the back surface	165	Vertebrae	Acc = 95%	Local Geometric Descriptors and SVM
Castro-Mateos, 2014 [20]	Feature Extraction and Segmentation	3D MRI	59	Discs	DICE = 88.4%	Statistical shape model space and B-Spline space
Raudner, 2020 [21]	Feature Extraction	MRI	58	Discs	/	GRAPPATINI
Abdollah, 2020 [22]	Feature Extraction	MRI	28	Discs, Vertebrae	/	Random Forest and texture analysis
Yang 2020 [8]	Feature Extraction and Classification	MRI	109	Discs	Acc = 88.3%	Gabor wavelet transformation and KLT feature tracker
Ruiz-España, 2015 [23]	Feature Extraction and Classification	MRI	67	Discs	Acc > 90%	Gradient Vector Flow, several ML models
Ketola, 2020 [24]	Feature Extraction and Classification	MRI	518	LBP	Acc = 83%	Texture feature extraction and Logistic Regression
Garcia-Cano, 2018 [10]	Feature Extraction and Regression	X-rays	150	Vertebrae	Cobb angle MAE = 4.79°	Independent component analysis and Random Forest

**Table 2:** Segmentation—Digital Image Processing. For each work, the main task is reported, whether it concerns only the segmentation of lumbar components, or if it aims to localize specific parts (e.g., the center of mass) of the components, or if it aims to identify each component (e.g., differentiating vertebrae between each other). If more structures are investigated, the correspondent results are reported in the same order in which structures are presented in the column “Structures involved”. Abbreviations are used for Magnetic Resonance Imaging (MRI), Computed Tomography (CT), Accuracy (Acc), Sensitivity (Sen), Specificity (Spec), Area Under the Curve (AUC), Gradient Vector Flow (GVF), Support Vector Machine (SVM).

Author/Year	Main Task	Data Type	# Patients	Structures Involved	Results	Model
Haq, 2015 [26]	Segmentation	3D MRI	21	Discs	DICE = 91.7%	Shape-aware models
Neubert, 2012 [27]	Segmentation and Identification	3D MRI	28	Discs and Vertebrae	DICE = 89 and 91%, Sen = 100%, Spec = 98%	Statistical shape model
Haq, 2020 [25]	Segmentation	CT images	18 SpineWeb	Discs	DICE = from 91,7 to 95,4%	Shape statistics deformable model
Li, 2018 [28]	Segmentation	CT images	115 (Microsoft R.+ SpineWeb)	Vertebrae	DICE = 92.1%	Gaussian Mixture Model + threshold
Ibragimov, 2017 [29]	Segmentation	CT images	30 vertebrae	Vertebrae	DICE = 84.7%	Landmark detection and deformable models
Yu, 2018 [30]	Segmentation	CT images	21 images	Vertebrae	DICE = 93.9%	Bone-sheetness assisted grid cut
Korez, 2015 [31]	Segmentation	CT images	220	Vertebrae	DICE = 94.6%	Shape-constrained deformable model
Al-Helo, 2011 [32]	Segmentation	CT images	50	Vertebrae	Visual evaluation	Active shape models and GVF-snake
Ruiz-España, 2015 [33]	Segmentation	CT images	10	Vertebrae	DICE = 95%	Selective Binary Gaussian Filtering Regularized Level Set
Huang, 2013 [34]	Segmentation	CT images	56	Vertebrae	DICE = 94%	Otsu thresholding, edge- and region-based level set
Mahdy, 2018 [35]	Segmentation and Localization	CT images	10	Vertebrae	Visual evaluation	Threshold and adaptive K-Means
Courbot, 2016 [36]	Localization	CT images	15	Vertebrae	Visual evaluation, Acc = 89.4%	Hidden Markov Chain segmentation
Rasoulian, 2013 [37]	Localization	CT images	32	Vertebrae	Visual evaluation, Center of mass MAE = 2mm	Multi-object shape model
Mastmeyer, 2006 [38]	Segmentation	CT images	41	Vertebrae	DICE > 98.6%	Volume growing and morphological operations
Jimenez-Pastor, 2020 [39]	Localization and Identification	CT images	272 images	Vertebrae	Localization error = 13.7mm, Acc = 74,8%	Decision forest + morphological image processing
Lee, 2011 [40]	Localization and Identification	CT images	19	Vertebrae	Localization error = 0.14mm, Acc = 93.2%	Threshold and thinning-based integrated cost
Klinder, 2009 [41]	Localization and Identification	CT images	64	Vertebrae	Localization error = 1.1mm, Acc = 92%	Triangulated shape models
Štern, 2009 [42]	Localization	MRI and CT images	13 and 29 images	Discs and Vertebrae	Localization error = 2.8 and 1.8 mm	Analysis of the geometry of spinal structures
Wong, 2008 [43]	Segmentation and Tracking	Fluoroscopic images	2 videos	Vertebrae	Visual evaluation	Wavelet and shape-active contour based
Zheng, 2011 [44]	Segmentation and 3D reconstruction	Fluoroscopic images	4	Vertebrae	Mean reconstruction error<1.6mm	Statistical shape models
Michopoulou, 2009 [45]	Segmentation	MRI	34	Discs	DICE = 90%	Atlas-robust-fuzzy C-Means
Fallah, 2018 [46]	Segmentation	MRI	50	Discs and Vertebrae	DICE = 92.5 and 91.4%	Hierarchical conditional random field and Random Forest
Ghosh, 2014 [47]	Segmentation	MRI	212	Discs and Vertebrae	DICE = 87 and 84%	Random Forest and context features
Kim, 2018 [48]	Segmentation	MRI	19	Vertebrae	DICE = 90%	Graph-based and line-based segmentation algorithms
Gaonkar, 2017 [49]	Segmentation	MRI	63	Vertebrae	DICE = 83%	Multi-parametric ensemble
Gawel, 2018 [50]	Segmentation	MRI	50	Vertebrae	DICE = 91.4%	Cascade classifier and Active Appearance Model
Engstrom, 2011 [51]	Segmentation	MRI	20	Muscles	DICE = 87%	Statistical shape model
Baum, 2018 [52]	Segmentation	MRI	10	Muscles	DICE = 83%	Average shape model and dual feature model
Zheng, 2004 [53]	Segmentation	Fluoroscopic images	1	Vertebrae	Visual evaluation	Hough transform and Fourier descriptors
Jurcak, 2008 [54]	Segmentation	MRI	20	Muscles	DICE = 77%	Probabilistic atlases and geodesic active contours
Fortin, 2017 [55]	Segmentation and Regression	MRI	30	Muscles	Reliability coefficient = 97-99%	Threshold
Neubert, 2013 [56]	Segmentation and Localization	MRI	44	Discs	DICE = 92.3%, AUC = 0.98	Active shape model, Linear Discriminant Analysis, SVM
Oktay, 2011 [57]	Localization and Identification	MRI	40	Discs	Localization rate = 95.4%, Acc = 97%	Probabilistic model and SVM
Castro-Mateos, 2016 [58]	Identification	MRI	48	Discs	Sensitivity = 87%	Active contour model and Feedforward NN
Kim, 2020 [59]	Localization	Ultrasound	50	Muscles	2mm discrepancy	Fuzzy C-Means Clustering
Lui, 2014 [60]	Localization	Ultrasound	10	Muscles	F1-Score = 90.9%	Decoupled Active Contour
Ribeiro, 2010 [61]	Segmentation	X-rays	41	Vertebrae	DICE = 91.7%	Gabor Filters and NN
Sa, 2016 [62]	Localization	X-rays	30	Vertebrae	True Positive Rate = 75%	GVF-snake and SVM

**Table 3:** Segmentation—Deep Learning. For each work, the main task is reported, whether it concerns only the segmentation of lumbar components, or if it aims to localize specific parts (e.g., the center of mass) of the components, or if it aims to identify each component (e.g., differentiating vertebrae between each other). If more structures are investigated, the correspondent results are reported following the same order by which structures are presented in the “Structures involved” column. Abbreviations are used for Magnetic Resonance Imaging (MRI), Computed Tomography (CT), Mean Absolute Error (MAE), Accuracy (Acc), Convolutional Neural Network (CNN), Support Vector Machine (SVM), Regression Trees (RT).

Author/Year	Main Task	Data Type	# Patients	Structures Involved	Results	Model
Iriondo, 2020 [63]	Segmentation	3D MRI	31	Discs	DICE > 85%	Coarse-to-fine context memory NN
Staartjes, 2021 [64]	Segmentation and Reconstruction	3D MRI	3	All structures	Visual evaluation	CNN
Lee, 2020 [65]	Segmentation and Reconstruction	CT images	280 images	All structures	MAE = 21 pixels	Generative Adversarial Networks
Fan, 2020 [66]	Segmentation and Reconstruction	CT images	108	All structures	Kambin triangle = 161 mm${}^{2}$	U-net
Malinda, 2020[67]	Segmentation	CT images	120	Vertebrae	DICE = 94.2%	Generative Adversarial Networks
Siemionow, 2020 [68]	Identification	CT images	45	Vertebrae	Acc = 96 to 99%	CNN
Netherton, 2020 [69]	Localization and Identification	CT images	330 images	Vertebrae	Localization error = 2.2 mm, Acc = 94%	X-net ensemble
Watanabe 2019 [70]	Regression	Moire images + X-rays	1996	Vertebrae	Cobb angle MAE = 3.42°	CNN
Kim, 2018 [71]	Segmentation	MRI	SpineWeb 20	Discs	DICE = 89.4%	CNN (BSU-net)
Shen, 2021 [72]	Segmentation	MRI	120	Discs, Spinal canal and Muscles	Jaccard: 87, 82 and 85%	Feedforward NN
Gaonkar, 2019 [73]	Segmentation	MRI	39295	Discs and Spinal canal	DICE = 88 and 87%	Discs: U-net, Canal: SVM and RT
Huang, 2020 [74]	Segmentation	MRI	100	Discs and Vertebrae	Jaccard = 92.6 and 94.7%	U-net
Li, 2021 [75]	Segmentation	MRI	120	Vertebrae and Spinal canal	DICE = 92.5%	CNN
Li, 2019 [76]	Segmentation	MRI	120	Muscles	DICE > 91.3%	Deformed U-net
Zhou, 2020 [77]	Segmentation	MRI	57	Vertebrae	DICE = 84.9%	U-net
Jamaludin, 2017 [78]	Classification	MRI	2009	Discs and Vertebrae	Acc = 95.6%	CNN
Natalia, 2020 [79]	Regression	MRI	515	Discs and Spinal canal	Mean error: 0.9 mm	SegNet and Contour Evolution Algorithm
Zhou, 2019 [80]	Identification	MRI	1318	Vertebrae	Acc = 98.9%	CNN
Forsberg, 2017 [81]	Identification	MRI with clinical notes	475	Vertebrae	Acc = 97%	CNN and parts-based graphical models
Baka, 2017 [82]	Identification	Ultrasound	19 data sets	Vertebrae	Acc = 92%	CNN and matching strategy
Cho, 2020 [83]	Segmentation and Regression	X-rays	629	Vertebrae	DICE = 82.1%, MAE = 8,055°	U-net
Li, 2016 [84]	Identification	X-rays	110	Vertebrae	Acc = 80.4%	CNN
Sa, 2017 [85]	Localization	X-rays	1081 images	Discs	Precision = 90.5%	Faster R-CNN

## Data Availability

No new data were created or analyzed in this study. Data sharing is not applicable to this article.

## Supplementary Materials

The following are available online at https://www.mdpi.com/article/10.3390/ijerph182010909/s1, Table S1: Summary of the methodological quality of included studies regarding the 4 domains assessing the risk of bias of the QUADAS-2 score; Table S2: Summary of the methodological quality of included studies regarding the 3 domains assessing applicability concerns of the QUADAS-2 score.
