# Supplementary material for: Artificial Intelligence and Computer Vision in Low Back Pain: A Systematic Review
Source: Int J Environ Res Public Health. 2021 Oct 17;18(20):10909. doi: 10.3390/ijerph182010909 (PMC8535895; doi:10.3390/ijerph182010909)
Supplement: Supplementary file 1 [file ijerph-18-10909-s001.zip › ijerph-1392391-SM.pdf]

Table S1: Summary of the methodological quality of included studies regarding the 4 domains assessing the **risk of bias** of the QUADAS-2 score.

Article	Patient selection	Index test	Reference standard	Flow and timing
Adankon, 2012	Unclear	Low	Low	Low
Castro-Mateos, 2014	High	Low	Low	High
Raudner, 2020	Low	Low	Low	Low
Abdollah, 2020	High	Low	Low	Low
Yang 2020	Low	Low	Low	Low
Ruiz-España, 2015	Low	Low	Low	Low
Ketola, 2020	High	Low	Unclear	Low
Garcia-Cano, 2018	High	Low	Low	High
Haq, 2015	Low	Low	Low	Low
Neubert, 2012	Unclear	Low	Low	Low
Haq, 2020	Low	Low	Low	Low
Li, 2018	Low	Low	Low	Low
Ibragimov, 2017	High	Low	Low	Low
Yu, 2018	Unclear	Low	Low	Low
Korez, 2015	Unclear	Low	Low	Low
Al-Helo, 2011	High	Low	Low	Low
Huang, 2013	Low	Low	Low	Low
Mahdy, 2018	Low	Low	Low	Low
Courbot, 2016	Low	Low	Low	Low
Rasoulian, 2013	Unclear	Low	Low	Low
Mastmeyer, 2006	Unclear	Low	Low	Low
Jimenez-Pastor, 2020	Low	Low	Low	Low
Lee, 2011	Unclear	Low	Low	Low
Klinder, 2009	High	Low	Low	High
Štern, 2009	Unclear	Low	Low	Low
Wong, 2008	Unclear	Low	Low	Low
Zheng, 2011	High	Unclear	Low	Low
Zheng, 2004	Unclear	Low	High	Low
Michopoulou, 2009	Unclear	Low	Low	Low
Fallah, 2018	High	Low	Low	Low
Ghosh, 2014	Low	Low	Low	Low
Kim, 2018	Low	Low	Low	Low
Gaonkar, 2017	Unclear	Low	Low	High
Gawel, 2018	Unclear	Low	Low	Low
Engstrom, 2011	Low	Low	Low	Low
Baum, 2018	Unclear	Low	Low	Low
Jurcak, 2008	Unclear	Low	Low	Low
Fortin, 2017	Low	Low	Low	Low
Neubert, 2013	High	Low	Low	High
Oktay, 2011	Unclear	Low	Low	Low
Castro-Mateos, 2016	Unclear	Low	Low	Low
Kim, 2020	Unclear	Low	High	Low
Lui, 2014	Unclear	Low	High	Low
Ribeiro, 2010	Unclear	Low	Low	Low
Sa, 2016	Unclear	Low	Low	Low
Iriondo, 2020	Unclear	Low	Low	High
Staartjes, 2021	High	Low	Low	Low
Lee, 2020	Unclear	Low	Low	Low
Fan, 2020	Low	Low	Low	Low
Malinda, 2020	Unclear	Low	Low	Low
Siemionow, 2020	Unclear	Low	Unclear	Unclear
Netherton, 2020	High	Low	Low	High
Watanabe 2019	Low	Low	Low	Low
Kim, 2018	Low	Low	Low	Low
Shen, 2021	Low	Low	Low	Low
Gaonkar, 2019	Unclear	Low	Low	Low
Huang, 2020	Low	Low	Low	Low
Li, 2021	Unclear	Low	Low	Low
Li, 2019	Unclear	Low	Low	Low
Zhou, 2020	Low	Low	Low	Low
Jamaludin, 2017	Low	Low	Low	High
Natalia, 2020	Unclear	Low	Low	Low
Zhou, 2019	Unclear	Low	Low	Unclear
Forsberg, 2017	Unclear	Low	Low	Low
Baka, 2017	High	Low	Low	High
Cho, 2020	High	Low	Low	Low
Li, 2016	Unclear	Low	Low	High
Sa, 2017	Unclear	Low	Low	Low

Table S2: Summary of the methodological quality of included studies regarding the 3 domains assessing **applicability** concerns of the QUADAS-2 score.

Article	Patient selection	Index test	Reference standard
Adankon, 2012	Low	Low	Low
Castro-Mateos, 2014	Low	Low	Low
Raudner, 2020	Low	Low	Low
Abdollah, 2020	Low	Low	Low
Yang 2020	Low	Low	Low
Ruiz-España, 2015	Low	Low	Low
Ketola, 2020	High	Low	Low
Garcia-Cano, 2018	High	Low	Low
Haq, 2015	Low	Low	Low
Neubert, 2012	Low	Low	Low
Haq, 2020	Low	Low	Low
Li, 2018	Low	Low	Low
Ibragimov, 2017	High	Low	Low
Yu, 2018	Low	Low	Low
Korez, 2015	Low	Low	Low
Al-Helo, 2011	High	Low	Low
Huang, 2013	Low	Low	Low
Mahdy, 2018	High	Low	Low
Courbot, 2016	High	Low	Low
Rasoulian, 2013	Unclear	Low	Low
Mastmeyer, 2006	Unclear	Low	Low
Jimenez-Pastor, 2020	High	Low	Low
Lee, 2011	Unclear	Low	Low
Klinder, 2009	High	Low	Low
Štern, 2009	Unclear	Low	Low
Wong, 2008	Unclear	Low	Low
Zheng, 2011	High	Low	Low
Zheng, 2004	Unclear	Low	High
Michopoulou, 2009	Unclear	Low	Low
Fallah, 2018	High	Low	Low
Ghosh, 2014	Unclear	Low	Low
Kim, 2018	High	Low	Low
Gaonkar, 2017	Unclear	Low	Low
Gawel, 2018	Low	Low	Low
Engstrom, 2011	High	Low	Low
Baum, 2018	High	Low	Low
Jurcak, 2008	High	Low	Low
Fortin, 2017	Low	Low	Low
Neubert, 2013	High	Low	Low
Oktay, 2011	Low	Low	Low
Castro-Mateos, 2016	Low	Low	Low
Kim, 2020	Unclear	Low	High
Lui, 2014	Unclear	Low	High
Ribeiro, 2010	High	Low	Low
Sa, 2016	Unclear	Low	Low
Iriondo, 2020	High	Low	Low
Staartjes, 2021	Unclear	Low	Low
Lee, 2020	Low	Low	Low
Fan, 2020	High	Low	Low
Malinda, 2020	Unclear	Low	Low
Siemionow, 2020	Unclear	Low	Unclear
Netherton, 2020	High	Low	Low
Watanabe 2019	High	Low	Low
Kim, 2018	Low	Low	Low
Shen, 2021	Low	Low	Low
Gaonkar, 2019	Unclear	Low	Low
Huang, 2020	Low	Low	Low
Li, 2021	Low	Low	Low
Li, 2019	Low	Low	Low
Zhou, 2020	Low	Low	Low
Jamaludin, 2017	Low	Low	Low
Natalia, 2020	Low	Low	Low
Zhou, 2019	Unclear	Low	Low
Forsberg, 2017	Unclear	Low	Low
Baka, 2017	High	Low	Low
Cho, 2020	Low	Low	Low
Li, 2016	Unclear	Low	Unclear
Sa, 2017	Unclear	Low	Unclear
